# Acutely kidney injury in the postoperative period of elective colorectal surgery: assessment of the age influence

**DOI:** 10.1590/0102-672020260000011e1940

**Published:** 2026-08-17

**Authors:** Leonardo Menezes GUARDA, Isaac José Felippe CORRÊA-NETO, Gabriela Gambi ROBLES, Victor Keniti Gomes NISHIYAMA, Rodrigo Ambar PINTO, Laercio ROBLES

**Affiliations:** 1 Faculdade Santa Marcelina, General Surgery – São Paulo (SP), Brazil.; 2 Hospital Santa Marcelina, Coloproctology Unit – São Paulo (SP), Brazil.; 3 Hospital Edmundo Vasconcelos, Urology Unit – São Paulo (SP), Brazil.; 4 Universidade de São Paulo, Medical College, Department of Gastroenterology – São Paulo (SP), Brazil.

**Keywords:** Acute Kidney Injury, Colorectal Surgery, Health of the Elderly, Postoperative Period, Injúria Renal Aguda, Cirurgia Colorretal, Saúde do Idoso, Período Pós-Operatório

## Abstract

**Background:**

The increase in life expectancy and the development of comorbidities bring a higher rate of complications and mortality in the postoperative period of surgical procedures in general. Among these associations, elective colorectal surgery and acute kidney injury are notable.

**Aims:**

To analyze risk factors associated with acute kidney injury in the postoperative period of elective colorectal surgeries in patients over 65 years of age. Additionally, to outline the epidemiological, clinical-surgical, and laboratory profile of the study population.

**Methods:**

A clinical, observational, longitudinal, prospective, and analytical study with a quantitative approach involving 30 patients from January 2023 to April 2024. Data analysis included pre, intra, and post-operative periods, with renal function assessment on the 3rd and 5th days after surgery.

**Results:**

The overall mean age was 70.4 years, 53.3% were female, 46.6% were smokers or former smokers, 63.3% had systemic arterial hypertension, and 23.3% had diabetes mellitus. Ten patients developed acute kidney injury in the postoperative period. The use of angiotensin-converting enzyme inhibitors and postoperative diuresis were significantly associated variables (p=0.039 and 0.034, respectively). The diuresis cutoff value to foretell non-progression to acute kidney injury was 0.5 mL/kg/h, with an accuracy of 88%, sensitivity of 100%, specificity of 77.78%, positive predictive value of 86.67%, and negative predictive value of 100%.

**Conclusions:**

In elderly patients undergoing elective colorectal surgery for oncological treatment, the incidence of acute kidney injury was high.

## ARTICLE HIGHLIGHTS

Analysis of risk factors associated with acute kidney injury in the postoperative period of elective colorectal surgeries in patients over 65 years of age.

One third of patients developed acute kidney injury in the postoperative period.

The use of angiotensin-converting enzyme inhibitors and postoperative diuresis were acute kidney injury variables (p=0.039 and 0.034, respectively).

The diuresis cutoff value to predict no progression to acute kidney injury was 0.5 mL/kg/h with an accuracy of 88%, sensitivity of 100%, specificity of 77.78%, positive predictive value of 86.67%, and negative predictive value of 100%.

## Summary

**CENTRAL MESSAGE:**

Acute kidney injury, also known as acute renal injury, is a syndrome resulting from an abrupt loss of kidney function due to decreased glomerular filtration rate. Postoperative acute kidney injury is associated with prolonged hospital recovery, increased healthcare costs, and higher mortality following major surgeries, complicating the perioperative period in up to 50% of these patients. Therefore, detecting acute kidney injury in its early stages, even before the onset of symptoms, especially in elderly patients, is essential for improving the management of this condition and reducing postoperative morbidity and mortality in elective colorectal surgery patients.

**PERSPECTIVES:**

The increased incidence of acute kidney injury in individuals over 60 years old may be attributed to a combination of factors, including comorbidities, nephrotoxic medications, and structural and functional changes associated with aging. Additionally, high anesthetic risk in elective surgeries, intraoperative complications, and pre-existing chronic kidney disease directly influence the development of acute kidney injury, especially in the elderly. In elderly patients undergoing elective colorectal surgery for oncological treatment, the incidence of Stage 1 acute kidney injury was high and associated with postoperative urine output and the non-use of angiotensin-converting enzyme inhibitors.

## VISUAL ABSTRACT

**Figure:**
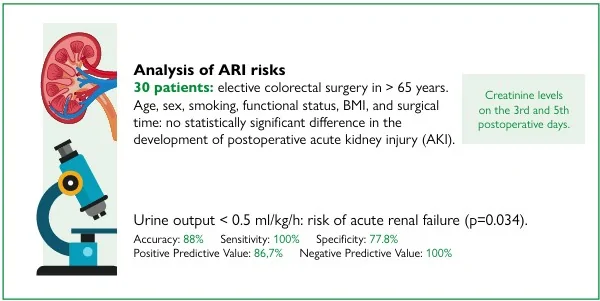

## RESUMO

**Racional:**

O aumento da expectativa de vida e o desenvolvimento de comorbidades trazem consigo uma maior taxa de complicações e mortalidades no pós operatório de procedimentos cirúrgicos em geral e, dentre essas associações, pode-se citar a cirurgia colorretal eletiva e a injúria renal aguda (IRA).

**Objetivos:**

Analisar fatores de riscos relacionados à IRA no pós-operatório de cirurgias colorretais eletivas em paciente com mais de 65 anos de idade. Além disso, traçar o perfil epidemiológico, clínico-cirúrgico e laboratorial da população do estudo.

**Métodos:**

Estudo clínico, observacional, longitudinal, prospectivo e analítico de caráter quantitativo de 30 pacientes no período de janeiro de 2023 a abril de 2024 com análise de dados de pré, intra e pós operatórios e avaliação da função renal no 3º e 5º dias após o procedimento cirúrgico.

**Resultados:**

A média de idade foi de 70,4 anos, 53,3% eram do sexo feminino, 46,6% eram tabagistas ou ex-tabagistas, 63,3% apresentavam hipertensão arterial sistêmica e 23,3% diabetes mellitus. Dez pacientes evoluíram com IRA no pós operatório e o uso de inibidores da enzima de conversão da angiotensina e a diurese no pós operatório foram variáveis relacionadas p=0,039 e 0,034, respectivamente. O valor de corte da diurese para predizer não evolução para IRA foi de 0,5 mL/Kg/h com uma acurácia de 88%, sensibilidade de 100%, especificidade de 77,78%, valor preditivo positivo de 86,67% e valor preditivo negativo de 100%.

**Conclusões:**

Em idosos submetidos a cirurgia colorretal eletiva para o tratamento oncológico, a incidência de injúria renal aguda mostrou-se elevada.

## INTRODUCTION

 Life expectancy has increased significantly from the past century to the present, whether due to reduced mortality, advances in science, vaccination coverage, medical care, a healthier lifestyle, or improved healthcare and hygiene practices^14^. Between 1990 and 2019, global life expectancy for both sexes increased from 64 years to 72.8 years^2^. 

 In Latin America, life expectancy at birth increased from 67.7 years in 1990 to 72.2 years in 2021^2^. In the European Union and North America, this figure rose from 73.6 years to 77.2 years, exemplifying a rapid and pronounced aging process in both developing and developed countries^1,14^. 

 The elderly population, defined by the World Health Organization (WHO) as individuals over 65 years of age, is also expected to experience significant demographic growth^1,6,8,12^. In 2022, this population accounted for 9.7% of the total, with projections estimating an increase to 16.4% by 2050^1^. Furthermore, by that same year, the number of individuals over 65 is expected to surpass the population of children under five years old and equal the number of those under 12 years of age^2^. 

 However, with increased longevity, the incidence of chronic diseases such as cancer, diabetes mellitus, systemic arterial hypertension, dementia, disabling diseases, pulmonary diseases, and kidney diseases has risen significantly^14^. 

 In this context, the increase in life expectancy and the development of comorbidities are associated with higher rates of complications and mortality in the postoperative period of surgical procedures in general^10^. Among these associations, elective colorectal surgery and acute kidney injury (AKI) stand out. 

 AKI, also known as acute renal injury (ARI), is a syndrome resulting from an abrupt loss of kidney function due to decreased glomerular filtration rate (GFR). It can be diagnosed and staged according to the Acute Kidney Injury Network (AKIN) classification^15^. According to these guidelines, AKI is defined as: an increase in serum creatinine by >0.3 mg/dL from baseline within 48 hours, a 50% increase in known baseline serum creatinine within the last seven days, and/or urine output <0.5 mL/kg/h for six hours^15^. 

 Postoperative AKI is associated with prolonged hospital recovery, increased healthcare costs, and higher mortality following major surgeries, complicating the perioperative period in up to 50% of these patients^5,13^. Despite its significant impact, AKI remains one of the most underdiagnosed and undertreated complications in hospitalized patients^5^. 

 Therefore, detecting AKI in its early stages, even before the onset of symptoms, especially in elderly patients, is essential for improving the management of this condition and reducing postoperative morbidity and mortality in elective colorectal surgery patients. 

 The objective of this study is to analyze risk factors associated with AKI in the postoperative period of elective colorectal surgeries in patients over 65 years of age. Additionally, to outline the epidemiological, clinical-surgical, and laboratory profile of the study population. 

## METHODS

 This is a clinical, observational, longitudinal, prospective, and analytical study with a quantitative approach. It was conducted at a university hospital in São Paulo with 30 patients undergoing elective colorectal surgery. The study was approved by the Ethics Committee of the Institution (number 6710444). 

 Patients included were those diagnosed with colorectal cancer who underwent elective surgery, aged over 65 years, of both sexes, hospitalized and operated on from January 2023 to April 2024, who either survived or died after the 5th postoperative day. Patients requiring reoperation before the 3rd postoperative day, those who did not sign the informed consent form (ICF), and those with incomplete medical records were excluded. 

 Data were collected using electronic medical records and organized and stored with Microsoft Excel. 

 The variables studied were:

- Preoperative data: age, sex, race, body mass index (BMI), performance status (Karnofsky Performance Status (KPS)), smoking, comorbidities, use of renin-angiotensin-aldosterone system inhibitors (angiotensin-converting enzyme inhibitors (ACEI)), weight loss >10% of body weight in six months, reason for surgery, type of surgery, anesthetic assessment (American Society of Anesthesiology (ASA)), preoperative creatinine, baseline GFR (evaluated by the Chronic Kidney Disease Epidemiology Collaboration (CKD-EPI)), preoperative total protein, and neoadjuvant therapy with radiotherapy and chemotherapy.
- Intraoperative data: surgical access route, blood loss, need for blood transfusion, volume administered, urine output, fluid balance, presence of intraoperative hypotension, surgical time, and use of antibiotics.
- Postoperative data: creatinine and clearance on the 3rd and 5th postoperative days, need for and type of ostomy, anastomotic fistula, postoperative complications, volume administered postoperatively, urine output, losses through drains and/or ostomies, use of nonsteroidal anti-inflammatory drugs (NSAIDs), use of vasoactive drugs, postoperative antibiotic use, and use of contrast for imaging exams.
- AKI classification: The diagnosis of AKI requires at least one of the following criteria:
  - Increase in creatinine by 0.3 mg/dL within 48 hours;
  - Creatinine greater than 1.5 times the baseline value or presumed within the last 7 days;
  - Urine output below 0.5 mL/kg/hour for 6 hours.

### AKI Stages

- Stage I: Increase in creatinine >0.3 mg/dL or 1.5 to 1.9 times the baseline value; urine output <0.5 mL/kg/hour for 6-12 hours.
- Stage II: Creatinine 2.0-2.9 times the baseline value; urine output <0.5 mL/kg/hour for >12 hours.
- Stage III: Creatinine >3 times the baseline value or >4 mg/dL, or need for dialysis; urine output <0.3 mL/kg/hour for >24 hours, or anuria for 12 hours.

If there is a discrepancy between serum creatinine data and urine output, the parameter indicating the most advanced stage should be used. 

### Statistical analysis

 Quantitative variables were described using descriptive statistics, including mean, median, standard deviation, and standard error, and the Shapiro-Wilk test was used to assess normality. Categorical variables were described using their absolute values in a 2x2 contingency table. 

 Comparisons between parametric and non-parametric variables were performed using the Student’s t-test and the Mann-Whitney U test, respectively. For categorical variables, the ꭓ^2^ test with Fisher’s correction was applied when necessary. Binary logistic regression, the stepwise method, and univariate and multivariate analyses were used to identify predictors of AKI. 

 Youden’s index was applied to determine the best postoperative urine output cutoff value to predict the group of patients likely to develop AKI after surgical intervention. The Receiver Operating Characteristic (ROC) curve was generated based on the determined urine output cutoff value, along with its corresponding sensitivity, specificity, positive predictive value, negative predictive value, and accuracy. 

 For all analyses, a p-value <0.05 was considered statistically significant. All analyses were performed using Jamovi software. 

## RESULTS

 A total of 30 patients who underwent elective colorectal surgery between June 2023 and March 2024 were analyzed. The overall mean age was 70.4 years (range: 66-85 years), with 53.3% being female and 46.6% current or former smokers. Regarding comorbidities, systemic arterial hypertension was present in 63.3% of cases, diabetes mellitus in 23.3%, dyslipidemia in 16.6%, hypothyroidism in 9.9%, heart failure in 6.6%, and chronic kidney disease in 3.3% of the patients. 

 Regarding the surgical procedures performed, 46.6% were rectosigmoidectomies, followed by right hemicolectomy (23.3%), left hemicolectomy (13.3%), transversectomy (6.6%), left proctocolectomy (6.6%), and abdominoperineal rectal amputation (3.3%). 

 Analyzing the variables of preoperative creatinine, creatinine levels on the 3rd and 5th postoperative days, and postoperative urine output, 10 patients (33.3%) developed AKI, with two of them (6.6%) classified as Stage II AKI. 

 Initially, 15 numerical variables were analyzed quantitatively, with mean, median, standard deviation, and standard error being calculated, as shown in Table 1. Table 1 compares patients who did not develop AKI with those who met the criteria for it. 

**Table 1:** Numerical variables (quantitative data) of patients undergoing elective colorectal surgery, organized into two general groups: with and without progression to AKI (0=without AKI; 1=with AKI).

Variables	Group	n	Mean	Median	Standard deviation	Standard error	p-value
Age	0	20	73.050	72.000	5.549	1.241	0.797*
	1	10	73.600	73.000	5.296	1.6746	
BMI	0	20	25.660	25.350	3.481	0.778	0.378†
	1	10	24.890	24.150	5.154	1.6297	
Preoperative creatinine	0	20	1.460	0.850	2.474	0.553	0.826†
	1	10	0.905	0.890	0.198	0.0627	
Preoperative glomerular filtration rate	0	20	74.330	82.900	26.984	6.034	0.962†
	1	10	77.833	75.000	21.237	7.0791	
Preoperative total protein	0	20	6.750	6.800	0.758	0.170	0.706*
	1	10	6.630	6.500	0.920	0.2910	
Creatinine levels on the 3rd postoperative day	0	20	1.390	0.845	2.461	0.550	0.113†
	1	10	1.278	1.135	0.590	0.1865	
Creatinine clearance on the 3rd postoperative day.	0	20	77.610	88.000	26.387	5.900	0.235†
	1	10	61.340	57.500	29.617	9.3656	
Creatinine levels on the 5th postoperative day	0	20	1.300	0.775	2.121	0.474	0.053†
	1	10	1.208	1.090	0.550	0.1741	
Creatinine clearance on the 5th postoperative day	0	20	79.630	93.850	28.717	6.421	0.116*
	1	10	62.230	54.850	25.374	8.0239	
Intraoperative volume	0	20	2280.00	2400.00	795.117	177.793	0.807†
	1	10	2495.00	2000.00	1519.585	480.5350	
Intraoperative urine output	0	20	368.000	400.000	204.697	45.772	0.092†
	1	10	368.500	175.000	627.632	198.4748	
Fluid balance	0	20	2004.50	1900.00	898.791	200.976	0.791†
	1	10	2126.50	1945.00	936.495	296.1457	
Fluid loss in the postoperative period	0	13	109.62	40.000	144.849	40.174	0.122†
	1	7	233.571	150.000	265.498	100.3489	
Surgery duration	0	20	3.400	3.000	1.142	0.255	0.765†
	1	10	4.100	3.000	3.143	0.9939	
Performance status (KPS)	0	20	89.50	90.00	9.445	2.112	0.250†
	1	10	85.00	90.00	10.801	3.4157	

Statistical test used to determine the p-value: *Student’s t-test; †Mann-Whitney U test.

BMI: body mass index; KPS: Karnofsky Performance Status.

Source: Elaborated by the authors.

 The Shapiro-Wilk test was used to assess whether the 15 quantitative variables in the table above are parametric, meaning they follow a normal distribution. It was demonstrated that the only parametric variables were age, preoperative total protein, and GFR on the 5th postoperative day. 

 In the second stage, 12 categorical variables were analyzed based on their qualitative data. Considering that 20 patients did not develop AKI and 10 patients did, this analysis used Pearson’s ꭓ^2^ test for comparison to obtain the p-value for each variable, as shown below Table 2. 

**Table 2:** Categorical variables (qualitative data) of patients undergoing elective colorectal surgery, organized into two general groups: with and without progression to acute kidney injury.

Variables	Group	Without AKI	With AKI	Total	p-value
Sex	Feminine	11	5	16	0.796
	Masculine	9	5	14	
Smoking	Non-Smoker	9	7	16	0.196
	Smoker	11	3	14	
Weight loss >10%	No	13	7	20	0.784
	Yes	7	3	10	
Ostomy	No	16	7	23	0.542
	Yes	4	3	7	
Neoadjuvant therapy with RT and/or CT	No	17	9	26	0.704
	Yes	3	1	4	
Fistula	No	18	9	27	1.000
	Yes	2	1	3	
Surgical approach	Laparotomy	16	10	26	0.129
	Laparoscopy	4	0	4	
Blood transfusion	No	17	9	26	0.704
	Yes	3	1	4	
Intraoperative hypertension	No	10	6	16	0.605
	Yes	10	4	14	
Use of vasoactive drugs	No	10	5	15	1.000
	Yes	10	5	15	
Use of contrast in the perioperative period	No	12	5	17	0.602
	Yes	8	5	13	
Use of ACEI in the preoperative period	No	6	8	14	0.010
	Yes	14	2	16	

AKI: acute kidney injury; RT: radiotherapy; CT: chemotherapy; ACEI: angiotensin-converting enzyme inhibitor.

Source: Elaborated by the authors.

 The initial analysis of categorical variables in contingency tables using Pearson’s Chi-square test showed that the use of ACEIs for systemic arterial hypertension had a significant p-value of 0.010, a relative risk of 0.490, and a 95% confidence interval (CI) of 0.260-0.922. Thus, a binary logistic regression test was performed to investigate which numerical and categorical variables could be predictors of AKI. The stepwise method was used to identify the best combination of variables that may predict AKI. 

 In a univariate analysis, preoperative ACEI use and postoperative urine output were identified as predictors of AKI Table 3. 

**Table 3:** Univariate analysis of the variables: preoperative angiotensin converting enzyme inhibitor use and postoperative urine output.

Variables	Univariate	
	OR (95%CI)	p-value
Use of ACEI	0.127 (0.0178 – 0.905)	0.039*
Postoperative urine output	9.27e-4 (1.44e-6 -0.594)	0.034*

* p<0.05.

OR: odds ratio; CI: confidence Interval; ACEI: angiotensin converting enzyme inhibitor.

Source: Elaborated by the authors.

 The likelihood of a patient using ACEIs in the preoperative period developing AKI up to the 5th postoperative day was 0.12 times that of those who did not use the drug, meaning the possibility of AKI in those who did not use ACEIs is 7.87 times (1/0.123) higher than in those who used the medication alone. Regarding diuresis, the cutoff value for predicting no progression to AKI was 0.5 mL/Kg/h with an accuracy of 88%, sensitivity of 100%, specificity of 77.78%, positive predictive value of 86.67%, and negative predictive value of 100%. 

## DISCUSSION

 Preventable postoperative complications in colorectal surgeries represent a challenge for patients, surgeons, and the healthcare system^11^. In the United States alone, approximately $41 billion is spent annually due to these complications^9^. Among them, AKI is the main one and poses several risk factors in different surgical contexts, including advanced age, emergency surgery, cirrhosis, obesity, peripheral vascular disease, chronic obstructive pulmonary disease, and especially chronic kidney disease, as well as intraoperative and postoperative management^5,11,16^. 

 The occurrence of AKI in non-cardiac and non-vascular surgeries is less studied due to its lower incidence^10^. However, according to the American College of Surgeons’ Surgical Quality Improvement Program, approximately 1% of general surgery cases develop AKI, leading to an eightfold increase in all-cause mortality within 30 days^3^. 

 A study conducted by Zorilla-Vaca et al.^16^ observed an overall incidence of postoperative AKI of 7.7% among 1,652 patients analyzed, with a higher incidence in those over 60 years old (13.8% vs. 5.13%). Additionally, it showed that independent risk factors for AKI were age over 60 years (CI 1.01-1.05), male gender, ASA III or IV, chronic kidney disease, surgical approach by laparotomy, and serum albumin <3.5 g/dL. 

 Similarly, Mannion et al.^7^ demonstrated that patients who developed AKI were significantly older, male, had a higher BMI, and had higher ASA scores. Additionally, these authors found an incidence of AKI of 24.6% in the postoperative period, a result similar to the present study. 

 Several studies have verified that one of the main risk factors for postoperative AKI is advancing age^4,5,9,16^, including the work of Biteker et al., which indicated that for each one-year increase in age, there is a 14% increase in the risk of developing AKI^2^. These data corroborate the importance of the present study in evaluating AKI in elderly patients following elective colorectal surgery. 

 The increased incidence of AKI in individuals over 60 years old may be attributed to a combination of factors, including comorbidities, nephrotoxic medications, and structural and functional changes associated with aging^5,11^. Additionally, high anesthetic risk in elective surgeries, intraoperative complications, and pre-existing chronic kidney disease directly influence the development of AKI, especially in the elderly^5^. Therefore, early detection of AKI depends on identifying patients with risk factors for its development in the preoperative, perioperative, and postoperative periods. 

 In the present study, it was observed that the likelihood of developing AKI in patients who did not use ACEIs was 7.87 times higher than in those who had previously used them, suggesting that ACEI use may act as a potential protective factor. However, there is no current evidence supporting the use of ACEIs as a protective factor against postoperative AKI. 

## CONCLUSIONS

 In elderly patients undergoing elective colorectal surgery for oncological treatment, the incidence of Stage 1 AKI was high and associated with postoperative urine output and the non-use of ACEI 

## Data Availability

The datasets generated and/or analyzed during the current study are available from the corresponding author upon reasonable request.
